# PDL1-driven self-amplifying drug homing with immune homeostasis restoration

**DOI:** 10.1016/j.mtbio.2026.103538

**Published:** 2026-08-10

**Authors:** Xiaoshuang Wang, Wanze Zhang, Chenxi Zhao, Di Wang, Yingyu Zhang, Jianfeng Wang, Xinyu Li, Jinbao Wang, Xuemei Han, Fuxin Xue, Linlin Liu, Zhaohui Tang

**Affiliations:** a China-Japan Union Hospital of Jilin University, Changchun, 130033, China; b Key Laboratory of Polymer Ecomaterials, Changchun Institute of Applied Chemistry, Chinese Academy of Sciences, Changchun, 130022, China; c The First Hospital of Jilin University, Changchun, 130000, China

**Keywords:** Drug delivery, ROS sensitive, Self-sustaining feedback, Immunogenic cell death, Tumor immune homeostasis

## Abstract

Antibody-decorated nanomedicines can selectively deliver drugs to tumors. However, their targeting efficiency diminishes as the density of the target antigen decreases. To address this limitation, this study developed a PDL1-driven, self-sustaining feedback drug-homing nanoparticle, termed aPDL1-PLG-DOX nanoparticles (aPDL1-P-DOX). This construction was prepared by loading doxorubicin (DOX) into a phenylboronic acid-bearing poly(_*L*_-glutamic acid) carrier responsive to reactive oxygen species (ROS) through boron-nitrogen coordination, followed by conjugation with an anti-PDL1 monoclonal antibody (aPDL1). The aPDL1 moiety directs nanoparticles to PDL1-expressing tumors, where elevated ROS levels trigger DOX release. In turn, residual tumor cells upregulate PDL1 expression in response to DOX, thereby enhancing subsequent recruitment of aPDL1-P-DOX and establishing a self-sustaining tumor-selective drug delivery cycle. This feedback-induced mechanism not only increases the specificity and persistence of DOX accumulation in tumors but also leverages co-delivered aPDL1 to mitigate DOX-associated immunosuppression and restore immune homeostasis. In a murine colon cancer model, this strategy achieved 98.7% inhibition of tumor growth and significantly prolonged survival, representing a promising approach for durable and precise cancer therapy.

## Graphical abstract

**Figure:**
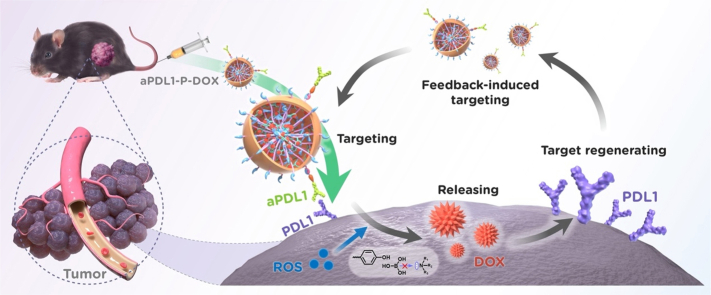

## 1 Introduction

Tumor-targeted nanomedicines functionalized with antibodies, peptides, aptamers, or small-molecule ligands have transformed systemic chemotherapy from a blunt cytotoxic approach into a precision-guided modality [[1], [2], [3], [4], [5]]. Antibody-drug conjugates (ADCs), exemplified by trastuzumab-emtansine, and peptide–drug conjugates have spearheaded this transformation, fueling a global market projected to exceed US $20 billion by 2030 [[6], [7], [8]]. Their clinical success stems from exquisite molecular recognition: ligands selectively engage receptors that are overexpressed or uniquely expressed by malignant cells, thereby delivering cytotoxic payloads across biological barriers while sparing healthy tissues [[9], [10], [11], [12]].

Despite steadily expanding clinical approvals, a fundamental limitation persists: most ligand-receptor interactions are non-renewable [[13], [14], [15]]. Tumors frequently down-regulate their cognate targets or acquire epitope mutations, rendering the delivery system obsolete [16]. Moreover, absolute target densities on tumor cells are modest. Even highly optimized ligands typically capture only a small fraction of the injected dose, leaving the majority of the therapeutic to circulate systemically [17,18].

Programmed death-ligand 1 (PDL1) represents a compelling exception to this “one-target, one-shot” paradigm [19,20]. PDL1 is widely upregulated across colorectal and many other solid tumors, where it suppresses antitumor immunity by engaging PD-1 on cytotoxic T cells [[21], [22], [23]]. Accordingly, anti-PDL1 antibodies that block this pathway have become a cornerstone of immuno-oncology [24]. From a drug-delivery perspective, the same PDL1 molecules that incapacitate immune responses could, in principle, be repurposed as docking sites for antibody-guided nanomedicines [21,25]. This duality presents a paradox: higher PDL1 abundance enhances drug targeting yet simultaneously entrenches immunosuppression [26]. Existing PDL1-directed ADCs have not resolved this conflict, as they either irreversibly deplete PDL1 or leave sufficient residual expression to sustain immune evasion [27]. A delivery system capable of dynamically amplifying PDL1 levels to reinforce its own targeting while concurrently neutralizing PDL1's immunosuppressive function would therefore represent a conceptual advance [28].

**Scheme 1:**
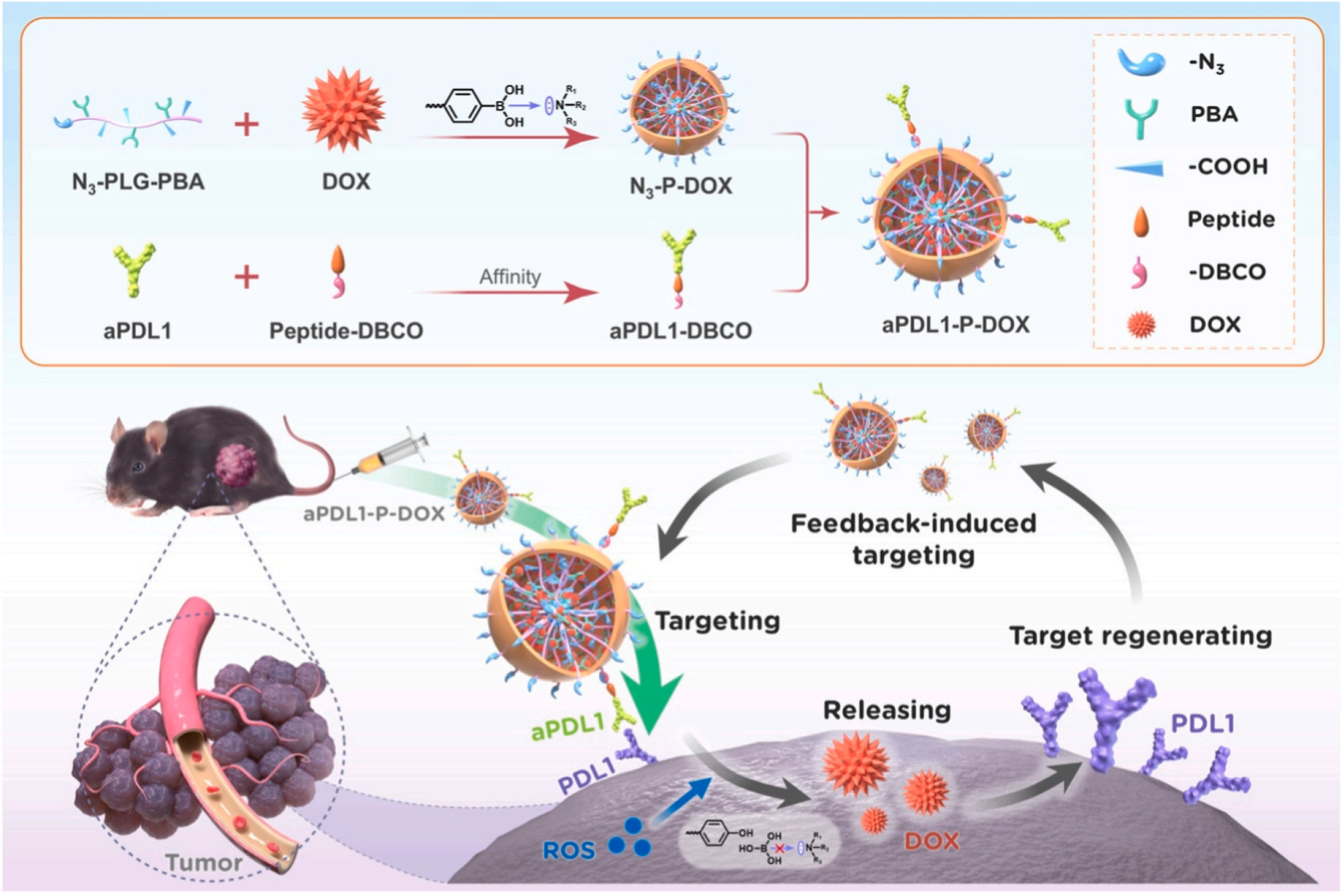
Schematic illustration of the multimodal tumor therapy mediated by aPDL1-P-DOX via a positive feedback mechanism. aPDL1-P-DOX actively targets tumors by specifically recognizing PDL1 on MC38 cells and selectively releases DOX in high ROS environments. DOX treatment induces upregulation of PDL1 at the tumor site, which recruits additional aPDL1-P-DOX, thereby amplifying therapeutic efficacy through a positive feedback loop during successive treatment cycles.

This paper proposes aPDL1-P-DOX, a self-amplifying nanoparticle system that converts PDL1 from a therapeutic liability into a therapeutic leverage point. The platform consists of doxorubicin-loaded polymeric micelles functionalized with an anti-PDL1 antibody via a high-affinity Fc-binding peptide. Upon arrival in the tumor microenvironment, elevated reactive oxygen species (ROS) trigger DOX release. Surviving tumor cells respond by upregulating PDL1, thereby expanding the number of available targets for subsequent nanoparticle recruitment [29]. This positive feedback loop results in a six-fold increase in intratumoral drug accumulation within 24 h. Concurrently, the anti-PDL1 coating blocks the newly induced PDL1 molecules, re-invigorating cytotoxic T cells and converting an immunosuppressive milieu into an immunostimulatory niche (Scheme 1). In murine MC38 colon carcinoma models, a single intravenous administration of aPDL1-P-DOX induced 98.7% tumor regression and yielded 80% long-term survival without detectable systemic toxicity. Beyond these quantitative gains, this research illustrates a general design principle: tumor self-defence pathways, exemplified by PDL1 upregulation, can be reprogrammed into autonomous therapeutic amplifiers. This feedback-enabled targeting paradigm may inspire the next generation of nanomedicines that exploit, rather than merely evade, the evolving biology of cancer.

## 2 Results and discussion

### 2.1 Characterization of aPDL1-P-DOX

**Fig. 1:**
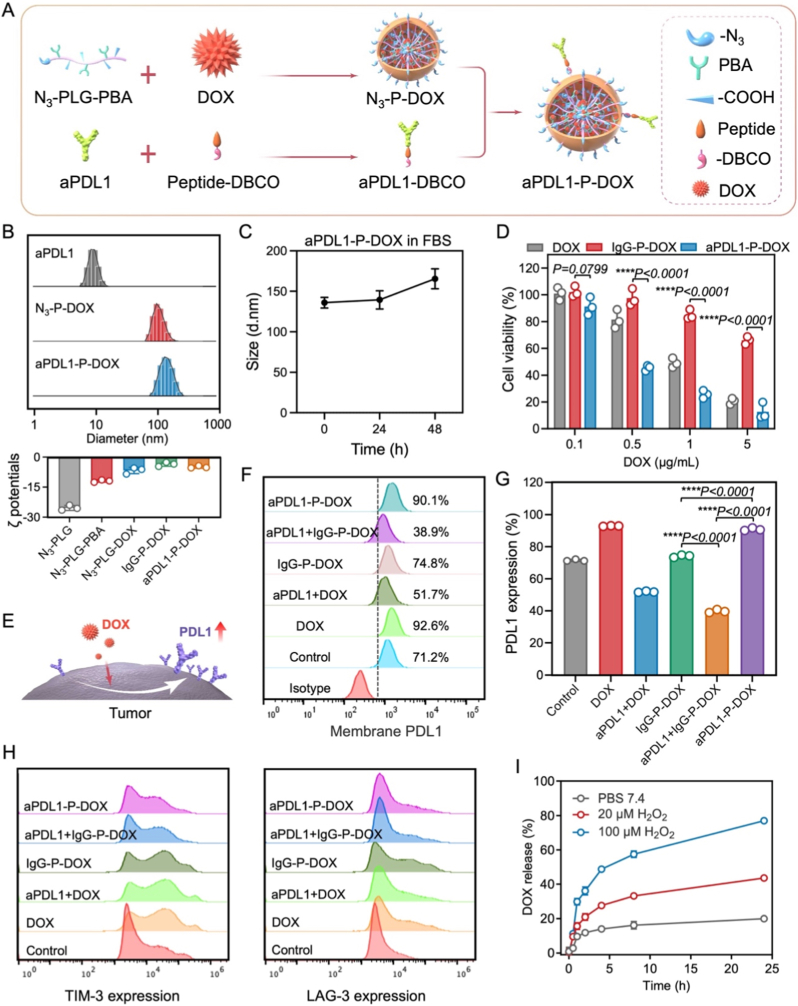
**Synthesis and characterization of aPDL1-P-DOX.** (A) Synthesis route of aPDL1-P-DOX. (B) Particle sizes and zeta potentials of aPDL1-P-DOX. (C) DLS of aPDL1-P-DOX incubated in FBS at 37°C for 48 h (n = 3). (D) Cell viability of MC38 cells after treatment with DOX, IgG-P-DOX, or aPDL1-P-DOX at various DOX concentrations for 48 h (n = 3). (E) Schematic illustration of DOX-induced PDL1 upregulation on tumor cells. (F) Representative flow cytometry plots and (G) quantitative analysis of PDL1 upregulation in MC38 cells (n = 3). (H) Representative flow cytometry plots of MC38 cells expressing surface TIM-3^+^ or LAG-3^+^ CTLs. (I) Release profile of DOX from aPDL1-P-DOX in PBS containing different concentrations of H_2_O_2_ (n = 3).

Peptide-PLG-DOX (Pep-P-DOX) was synthesized via a click reaction between the Dibenzocyclooctyne (DBCO) moiety on DBCO-Fc and the azide group of N_3_-PLG-DOX (N_3_-P-DOX). To construct aPDL1-PLG-DOX nanoparticles (aPDL1-P-DOX), Pep-P-DOX and aPDL1 were incubated in phosphate-buffered saline (PBS) under stirring for 4 h, which relies on the high binding affinity between the Fc-III-4C peptide on Pep-P-DOX and the Fc domain of aPDL1 (Fig. 1A). The ^1^H NMR spectra of N_3_-PLG-PBA and N_3_-P-DOX are displayed in Figure S1 and Figure S2, respectively.

Zeta potential measurements showed that N_3_-PLG, N_3_-PLG-PBA, N_3_-P-DOX, IgG-P-DOX and aPDL1-P-DOX possessed zeta potentials of −24.5 ± 2.5 mV, −12.0 ± 0.6 mV, −6.7 ± 1.6 mV, −3.6 ± 1.0 mV and −5.0 ± 0.6 mV, respectively (Fig. 1B), which can be attributed to abundant carboxyl groups on the polyglutamic acid backbone. Dynamic light scattering (DLS) characterization revealed that aPDL1-P-DOX had a hydrodynamic diameter of 156.5 ± 2.4 nm with a polydispersity index (PDI) of 0.2, while N_3_-P-DOX exhibited a smaller size of 102.3 ± 3.4 nm (PDI = 0.3), and free aPDL1 antibody was only 9.0 ± 0.5 nm. No residual free antibody was detected after assembly, verifying the successful fabrication of aPDL1-P-DOX nanocomplexes (Fig. 1B). Long-term stability assays demonstrated negligible particle size variation within 48 h of incubation in FBS and 6 days of incubation in PBS, confirming favorable storage stability of the nanoparticles (Fig. 1C and Fig. S3). Size distribution of aPDL1-P-DOX incubated with 100 μM H_2_O_2_ for 2 h compared to the native state. The increase in hydrodynamic diameter (∼400 nm vs. ∼120 nm) confirms the H_2_O_2_-triggered morphological changes of the nanoparticles (Fig. S4). ROS oxidizes phenylboronic acid into phenol and boric acid small molecules, which detach from the polymer backbone and eliminate B-N coordination bonds, thereby triggering DOX release. The loss of hydrophobic DOX from the inner core disrupts the core-shell structure, inducing polymer chain relaxation and significant particle size expansion.

The DOX encapsulation efficiency was determined by comparing the DOX content in purified N_3_-P-DOX with the initial feeding amount of DOX, and the value was calculated to be 78.5 ± 3.2% (n = 3). The drug loading content (DLC) was defined as the weight ratio of loaded DOX to the total polymer mass, which was 12.8 ± 0.6% (w/w, n = 3). In addition, the antibody conjugation efficiency was measured via BCA protein assay by comparing the free aPDL1 after ultrafiltration centrifugation amount coupled on purified aPDL1-P-DOX with the initially input aPDL1, achieving a conjugation efficiency of 89.3 ± 4.1% (n = 3), which was consistent with the results of our previous work [30].

### 2.2 In vitro antitumor activity and positive feedback validation

CCK-8 assays were performed to quantify the in vitro cytotoxicity of free DOX, IgG-P-DOX and aPDL1-P-DOX against MC38 colon cancer cells. All DOX-containing formulations elicited dose-dependent tumor cell killing (Fig. 1D). At equivalent DOX concentrations, aPDL1-P-DOX exhibited remarkably stronger antiproliferative effects than free DOX and IgG-P-DOX. This superior cytotoxicity originates from PDL1-mediated active tumor targeting, which facilitates massive intracellular nanoparticle accumulation and enhances chemotherapeutic damage to MC38 cells [31,32] We further investigated the DOX-mediated PDL1 upregulation effect to validate the in vitro positive feedback loop (Fig. 1E). MC38 cells were treated with free DOX or aPDL1-P-DOX separately. Flow cytometry results indicated that free DOX moderately induced PDL1 expression, whereas aPDL1-P-DOX treatment resulted in far more pronounced PDL1 overexpression compared with the untreated control group (Fig. 1F–G and Fig. S5), a phenomenon stemming from the active targeting property of conjugated aPDL1. On this basis, we proposed a self-amplifying positive feedback mechanism for this nanoplatform. Notably, flow cytometric quantification was restricted to viable MC38 cells by excluding dead cell populations, which proves that DOX-induced PDL1 elevation is an intrinsic adaptive response of surviving tumor cells, rather than a passive byproduct of cell apoptosis.

**Fig. 2:**
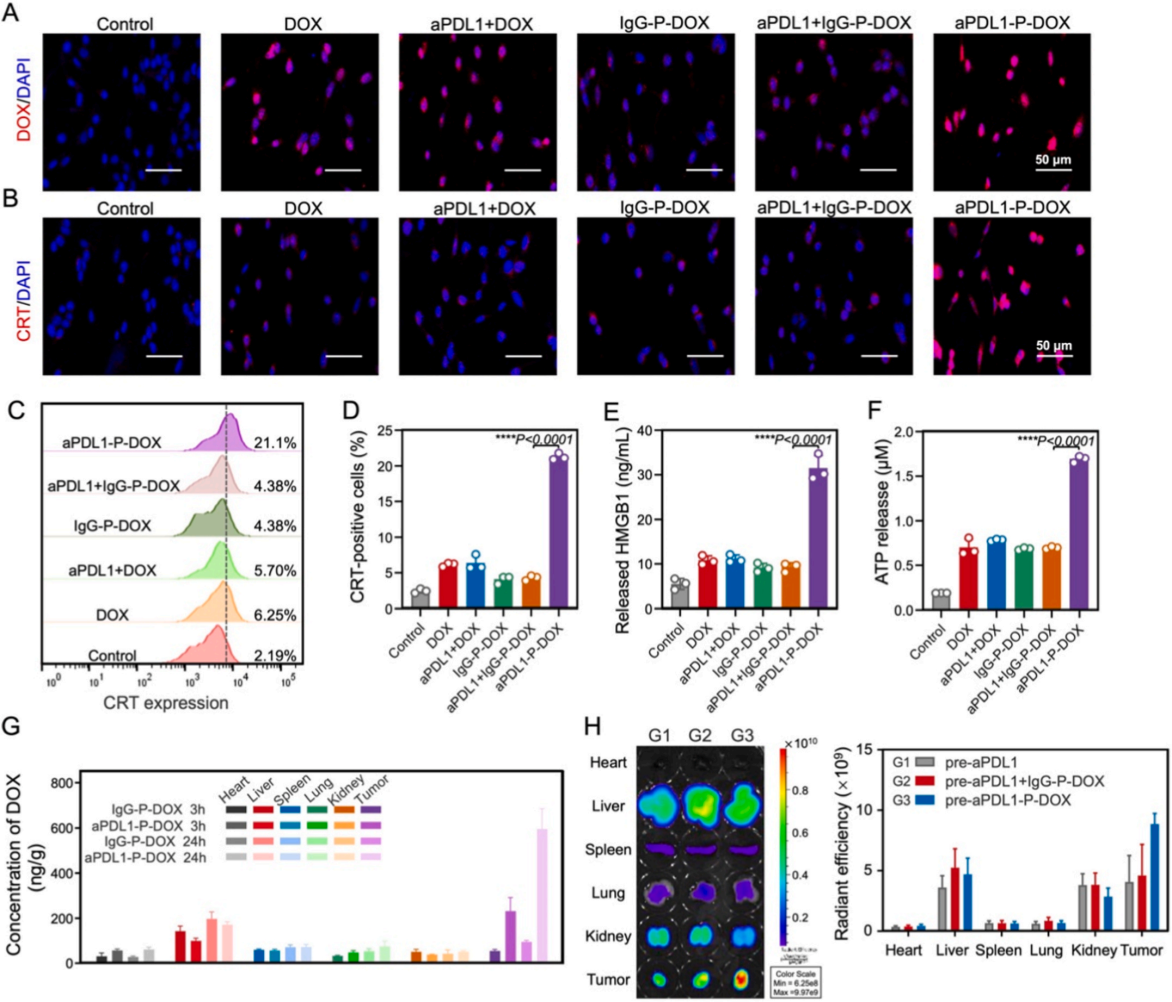
**Evaluation of the PDL1-mediated positive feedback effect of aPDL1-P-DOX *in vitro*. MC38 cells were treated with PBS, DOX, aPDL1+DOX, IgG-P-DOX, aPDL1+IgG-P-DOX or aPDL1-P-DOX for 24 h.** (A) Assessment of cellular uptake of DOX-based formulations mediated by the positive feedback mechanism (scale bar = 50 μm). (B) CLSM images of CRT expression in MC38 cells (scale bar = 50 μm). (C) Representative flow cytometry plots and (D) quantification of the population of MC38 cells expressing surface CRT (n = 3). (E) Extracellular HMGB1 levels released from MC38 cells (n = 3). (F) Extracellular ATP concentrations (n = 3). (G) HPLC quantification of IgG-P-DOX and aPDL1-P-DOX at 3 h and 24 h post-injection. (H) *In vitro* fluorescence imaging and quantitative fluorescence analysis of major organs and tumor tissues at 24 h (n = 3).

Confocal laser scanning microscopy (CLSM) was utilized to visualize and compare cellular uptake efficiency across distinct treatment groups. After 24 h incubation, MC38 cells receiving aPDL1-P-DOX displayed drastically stronger DOX-derived red fluorescence signals relative to cells treated with free DOX, free aPDL1 combined with free DOX, IgG-P-DOX, or free aPDL1 combined with IgG-P-DOX (Fig. 2A and Fig. S6). Collectively, these data confirm that surface-conjugated aPDL1 endows nanoparticles with specific active targeting capacity toward PDL1-positive MC38 cells (Fig. S7). Meanwhile, DOX delivered intracellularly further stimulates tumor cell PDL1 expression, which in turn recruits more aPDL1-P-DOX nanoparticles to tumor sites, forming a self-sustaining positive feedback cascade that continuously improves tumor-selective drug delivery.

### 2.3 In vitro immunogenic cell death (ICD) effect of aPDL1-P-DOX

To examine the ICD effect elicited by aPDL1-P-DOX in MC38 cells, key ICD hallmarks, including surface exposure of calreticulin (CRT) and extracellular release of HMGB-1 and Adenosine triphosphate (ATP), were evaluated. CLSM images revealed the strongest CRT signal on the surface of MC38 cells treated with aPDL1-P-DOX, indicating robust ICD induction (Fig. 2B). Flow cytometry confirmed that the proportion of MC38 cells expressing surface CRT was significantly higher in the aPDL1-P-DOX group compared to DOX, aPDL1+DOX, IgG-P-DOX, aPDL1+IgG-P-DOX, and the PBS control group (Fig. 2C and D). Extracellular HMGB-1 release, quantified via ELISA, was maximal in the aPDL1-P-DOX group, consistent with enhanced cellular uptake facilitated by PDL1-targeting (Fig. 2E). Similarly, extracellular ATP levels were significantly elevated following aPDL1-P-DOX treatment relative to other groups (Fig. 2F). Collectively, these results demonstrate that aPDL1-P-DOX potentiates DOX-mediated ICD through its self-amplifying feedback mechanism, thereby promoting anti-tumor immune responses.

### 2.4 Pharmacokinetic and biodistribution analysis

Pharmacokinetic analysis was performed in SD rats following intravenous administration of aPDL1-P-DOX. The pharmacokinetic curve (Fig. S8) indicated a prolonged circulation half-life of 28.4 ± 2.2 h for aPDL1-P-DOX, compared to 6.4 ± 1.4 h for N_3_-P-DOX. Full pharmacokinetic parameters of aPDL1-P-DOX and N_3_-P-DOX calculated by non-compartmental analysis (PKSolver) are summarized: “aPDL1-P-DOX possessed a longer t_1/2_ (6.42 h vs 3.21 h), higher AUC_0-inf_ (38.43 μg h/mL vs 13.33 μg h/mL) and mean residence time (7.70 h vs 3.12 h), as well as lower clearance (0.26 L/h/kg vs 0.75 L/h/kg) relative to N_3_-PLG-DOX. These results highlight the effect of aPDL1 conjugation on systemic retention.

The biodistribution of aPDL1-P-DOX was evaluated by administering an equivalent dose of IgG-P-DOX or aPDL1-P-DOX to MC38 tumor-bearing mice (tumor volume: 200 mm^3^). The mice were sacrificed at scheduled time points, and DOX content was measured in key organs and tumors. aPDL1-P-DOX accumulation in tumor tissue was 4.2-fold higher than IgG-P-DOX at 3 h post-administration and increased to 6.3-fold at 24 h (Fig. 2G). When tumor-bearing mice were intravenously injected via the tail vein 24 h in advance with aPDL1 (Pre-aPDL1), aPDL1+IgG-P-DOX (Pre-aPDL1+IgG-P-DOX), or aPDL1-P-DOX (Pre-aPDL1-P-DOX), followed by an injection of Cy5-labeled aPDL1-P-DOX (aPDL1-P-DOX/Cy5), the Pre-aPDL1-P-DOX group exhibited the strongest fluorescence enrichment (Fig. 2H). This observation supports the conclusion that DOX induces PDL1 regeneration in this project. The targeting capability of aPDL1-P-DOX and its induction of PDL1 regeneration promoted the tumor accumulation of aPDL1-P-DOX/Cy5. These findings substantiate that the self-sustaining feedback mechanism of aPDL1-P-DOX significantly enhances tumor-specific drug delivery and accumulation of aPDL1-P-DOX.

### 2.5 *In vivo* antitumor efficacy and immune responses of aPDL1-P-DOX

**Fig. 3:**
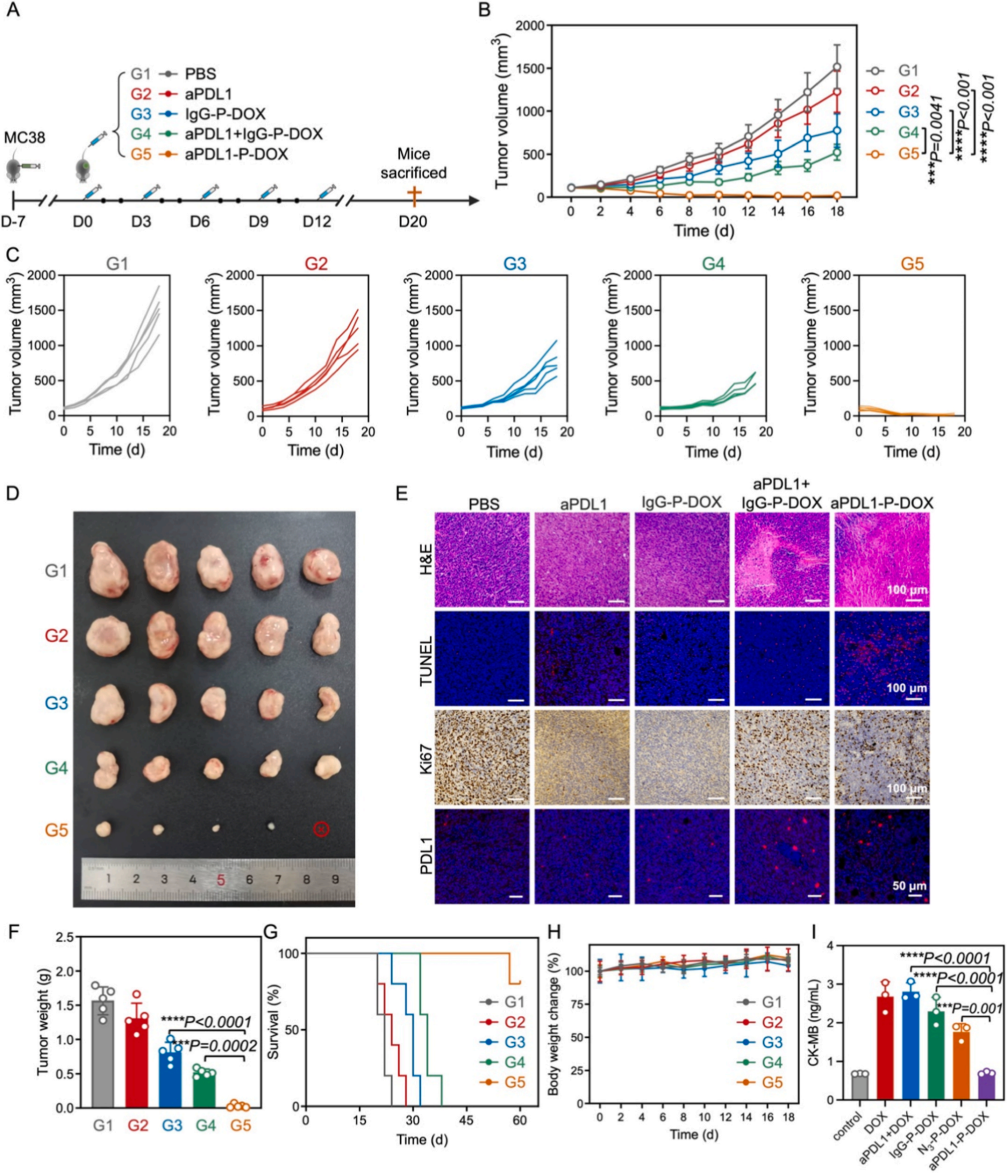
***In vivo* antitumor efficacy and immune activation of aPDL1-P-DOX in an MC38 model.** (A) Schematic diagram of the treatment regimen. (B) and (C) Tumor growth curves following various treatments (n = 5). (D) Representative tumor photographs at the endpoint (n = 5). (E) Staining of tumor sections post-treatment (scale bar = 100 μm). H&E, TUNEL, Ki67 and PDL1 staining of tumor sections subjected to different treatments. (F) Tumor weight of mice following various treatments (n = 5). (G) Survival analysis of mice within 60 days (n = 5). (H) Body weights at the endpoint (n = 5). (I) CK-MB ELISA assay of mice after different treatment.

The antitumor efficacy and immunomodulatory effects of aPDL1-P-DOX were investigated in MC38 tumor-bearing mice, following the treatment schedule depicted in Fig. 3A. As shown in Fig. 3B–D and F, tumors in the PBS group exhibited rapid growth, reaching approximately 1800 mm^3^ by day 20. In contrast, aPDL1-P-DOX treatment resulted in profound tumor growth inhibition, with an average tumor volume of 25.8 mm^3^ on day 20, corresponding to a tumor inhibition rate of 98.7%. Survival analysis (Fig. 3G) revealed that 80% of mice treated with aPDL1-P-DOX survived to day 60, whereas all mice in the other groups succumbed earlier, with the shortest survival observed in the PBS group. No significant body weight changes were observed across treatment groups, suggesting favorable tolerability and minimal systemic toxicity (Fig. 3H). Meanwhile, the CK-MB assay (Fig. 3I) showed no significant differences between aPDL1-P-DOX and control group.

Hematoxylin and eosin (H&E) staining showed extensive necrosis in aPDL1-P-DOX-treated tumors versus intact PBS controls (Fig. 3E). TUNEL, Ki67 and PDL1 staining confirmed apoptosis, with aPDL1-P-DOX group exhibiting reduced blue (viable) and enhanced red (apoptotic) fluorescence. Meanwhile, immunofluorescence staining further validated the upregulation of Cytotoxic T lymphocytes (CTLs) in the aPDL1-P-DOX group. Moreover, PDL1 expression was upregulated in tumor tissues following aPDL1-P-DOX treatment relative to the PBS group, suggesting the engagement of a positive feedback loop that potentiates the antitumor efficacy of aPDL1-P-DOX. We measured the levels of heat shock proteins HSP70 and HSP90 in the DAMP molecules of the tumors of the treated mice. We found that both proteins significantly increased (Fig. S9), with the highest level after aPDL1-P-DOX treatment.

**Fig. 4:**
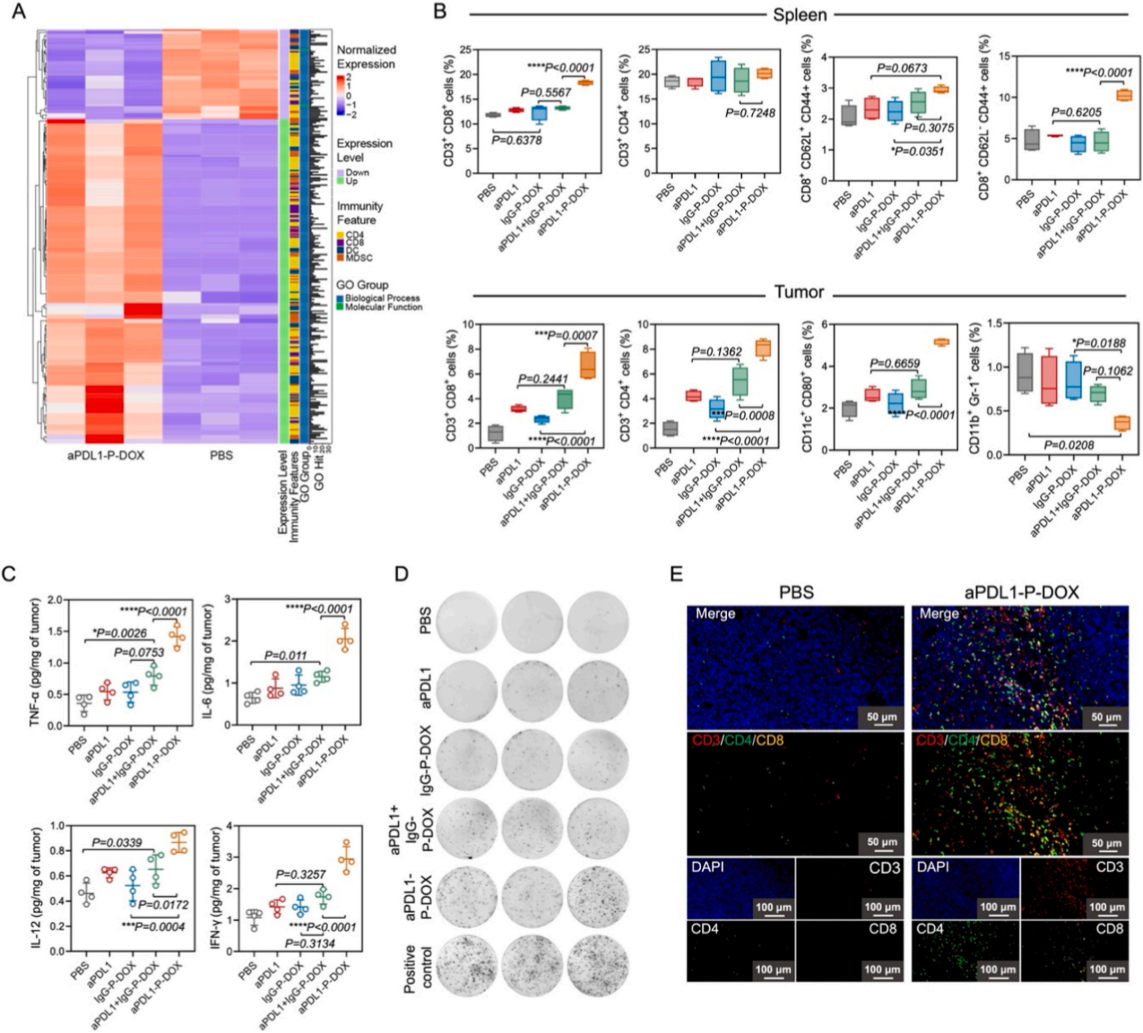
**Immune responses elicited by aPDL1-P-DOX treatment.** (A) Heatmap and bar plot illustrating expression and fold changes of selected immune-related genes across groups (aPDL1-P-DOX vs. PBS). (B) Flow cytometric analysis of tumor-infiltrating immune cells after 20 days of treatment, including CTLs (CD3^+^CD8^+^), Ths (CD3^+^CD4^+^), DCs (CD11c^+^CD80^+^), and MDSCs (CD11b^+^Gr-1^+^) in tumors, as well as CTLs (CD3^+^CD8^+^), Ths (CD3^+^CD4^+^), Tcm (CD3^+^CD8^+^CD62L^+^CD44^+^), and Tem (CD3^+^CD8^+^CD62L^−^CD44^+^) in spleens (n = 4). (C) Tumor cytokine levels of TNF-α, IL-6, IL-12, and IFN-γ determined by ELISA (n = 4). (D) IFN-γ ELISpot analysis following different treatments (PBS, aPDL1, IgG-P-DOX, aPDL1+IgG-P-DOX, and aPDL1-P-DOX) and positive control (n = 3). (E) Tumor sections from mice treated with PBS or aPDL1-P-DOX were stained with DAPI (blue), anti-CD3 (red), anti-CD4 (green) and anti-CD8 (yellow). (For interpretation of the references to color in this figure legend, the reader is referred to the Web version of this article.)

To evaluate antitumor immune activation, tumor-infiltrating lymphocytes and key cytokines were quantified. Compared with the PBS and aPDL1+IgG-P-DOX groups, aPDL1-P-DOX treatment significantly increased the proportion of tumor-infiltrating CTLs (6.6%) and Ths (8.2%) within the tumor, indicating robust local immunostimulation (Fig. 4A and B).

Interpretation of key upregulated genes (Cxcl1, Cxcl2, Cxcl10, S100a8, S100a9, Socs3, Tnfaip3, Nos2) in the context of immune cell recruitment and activation; functional implications of enriched GO terms (immune system processes, cytokine signaling, chemokine activity); and mechanistic insights from KEGG pathway enrichment (Cytokine-cytokine receptor interaction, NF-κB signaling, TNF signaling). DC infiltration was also elevated (5.2%), suggesting enhanced antigen presentation and T cell priming (Fig. 4B). MDSCs were markedly reduced in the aPDL1-P-DOX group (0.4%) compared to the PBS group (0.9%), reflecting effective alleviation of the immunosuppressive tumor microenvironment (Fig. 4B). Systemic immune responses were further evaluated using flow cytometry. The results revealed the highest levels of CTLs (18.4%), Tcm (2.9%), and Tem (10.2%) in the spleen following aPDL1-P-DOX treatment, demonstrating potent systemic tumor-killing activity and durable immune memory (Figure S10-11). Meanwhile, elevated levels of TNF-α, IL-6, IL-12, and IFN-γ were detected in the aPDL1-P-DOX group relative to the other groups (Fig. 4C), corroborated by ELISpot assays showing significant IFN-γ activation (Fig. 4D). Tumor sections from mice treated with PBS or aPDL1-P-DOX were stained with DAPI (blue), anti-CD3 (red), anti-CD4 (green) and anti-CD8 (yellow). Representative immunofluorescence images show that aPDL1-P-DOX treatment significantly enhanced the infiltration of CD3^+^CD8^+^ T cells into the tumor microenvironment compared to the PBS control group (Fig. 4E).

**Fig. 5:**
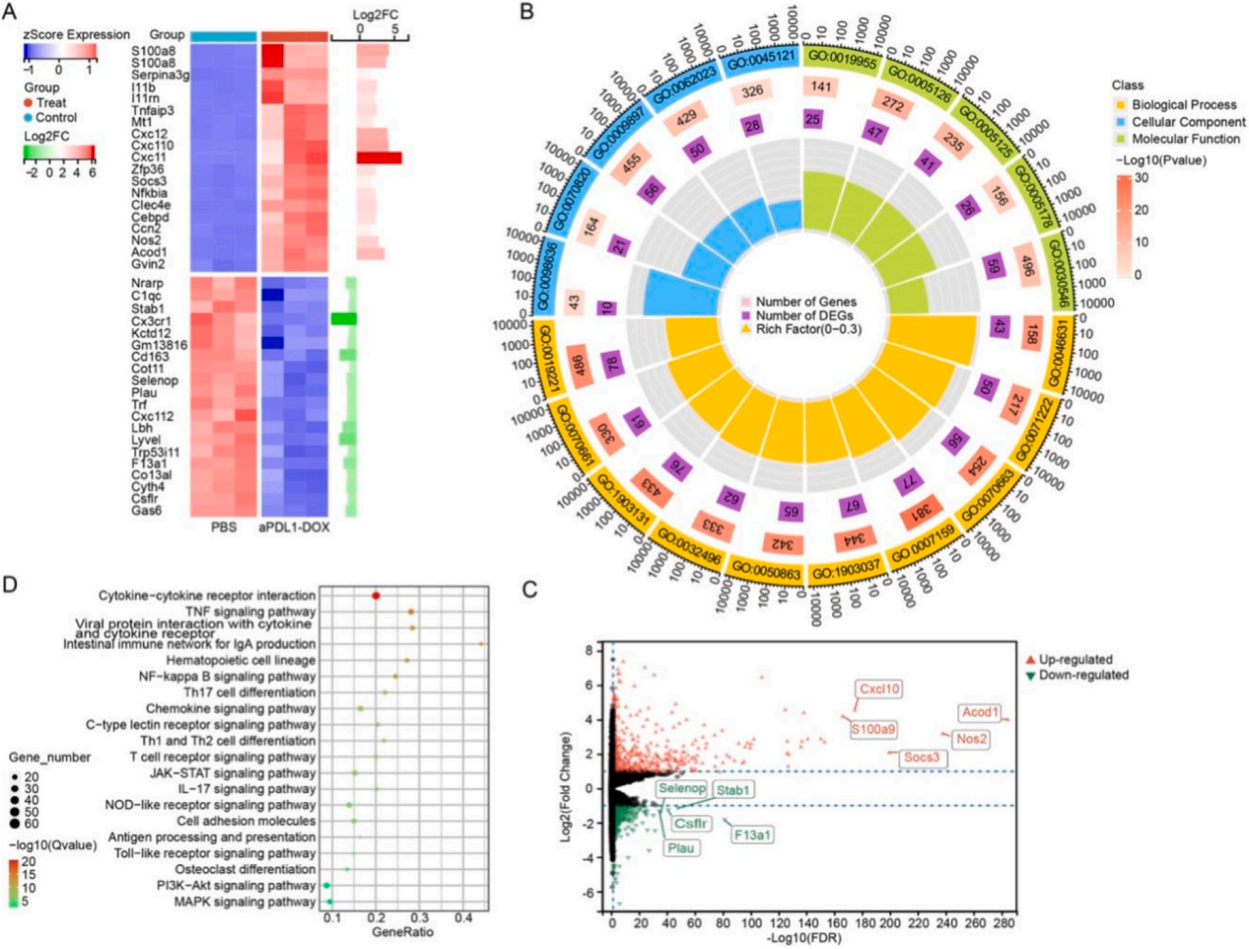
**Transcriptomic profiling reveals aPDL1-P-DOX remodels the tumor immune microenvironment through activation of key signaling pathways.** (A) Heatmap and bar plot show the expression and fold change of selected immune-related genes across groups (PBS and aPDL1-P-DOX). Colors represent scaled expression and log2 fold change values (top) (n = 3). (B) GO-related gene count of PBS, aPDL1+IgG-P-DOX and aPDL1-P-DOX (n = 3). (C) On day 3 after treatment, control and aPDL1-P-DOX-treated tumor tissues were taken for transcriptome sequencing. The overall distribution of regulated genes is represented by a volcano plot, in which the abscissa and ordinate represent the fold change of gene expression in different samples and statistical significance (p value) of the difference in gene expression, respectively. The red and blue dots represent the upregulated, down regulated and no significant changes of genes, respectively (n = 3). (D) The significance of KEGG pathway-related genes in the DOX pathway and PDL1 in tumors treated with PBS and aPDL1-P-DOX (n = 3). (For interpretation of the references to color in this figure legend, the reader is referred to the Web version of this article.)

Overall, these results demonstrate that aPDL1-P-DOX treatment not only achieves superior tumor growth inhibition but also robustly activates both local and systemic antitumor immune responses. This robust T-cell infiltration and activation was further corroborated by transcriptomic analysis, which revealed a broad upregulation of genes associated with cytokine signaling and immune response (Fig. 5).

Unsupervised hierarchical clustering of significantly differentially expressed genes (DEGs) clearly segregates the aPDL1-PDOX treatment group from the PBS control group (Fig. 5A). The heatmap demonstrates a widespread upregulation (indicated in red) of a cohort of genes critical for immune activation in the aPDL1-PDOX cohort. Key upregulated genes include pro-inflammatory chemokines (Cxcl1, Cxcl2, Cxcl10), markers of immune cell recruitment and activation (S100a8, S100a9), immunoregulatory genes (Socs3, Tnf aip3), and mediators of inflammatory signaling (Nos2). This pattern indicates a robust shift in the tumor microenvironment towards an immunostimulatory state. Gene Ontology (GO) enrichment analysis categorized the biological functions of the differentially expressed genes into three standard ontologies, Biological Process, Cellular Component, and Molecular Function (Fig. 5B). The analysis revealed a significant enrichment of terms associated with immune system processes, response to cytokine stimuli, and chemokine activity. The high rich factor for these terms indicates that the DEGs are specifically and robustly associated with these pro-inflammatory and immune-activating biological functions, underscoring a substantial shift in the tumor microenvironment following treatment. The volcano plot (Fig. 5C) provides a global view of the transcriptomic alterations, plotting the statistical significance (-log10(FDR)) against the magnitude of change (log2 Fold Change). It confirms a substantial number of statistically significant DEGs (FDR < 0.05) induced by aPDL1-P-DOX treatment. The plot highlights several of the most significantly upregulated genes, such as Cxcl10, Acod1(Irgl), Socs3, and Nos2, which are key players in cytokine signaling and macrophage-mediated immunity.

Complementarily, the Kyoto Encyclopedia of Genes and Genomes (KEGG) pathway enrichment analysis further elucidated the underlying signaling mechanisms (Fig. 5D). The results are summarized in a dot plot where the size of each dot represents the number of DEGs mapped to a given pathway, and the color corresponds to the statistical significance (-log10(P-value)). The aPDL1-P-DOX treatment induced a significant enrichment in pivotal signaling pathways, most notably the Cytokine-cytokine receptor interaction, NF-kappa B signaling pathway, and TNF signaling pathway. This finding confirms that the observed global gene expression changes are coordinately regulated through well-defined pro-inflammatory and immunomodulatory signal transduction cascades, highlighting the potential mechanism of action for the therapeutic regimen.

Collectively, the integrated transcriptomic data demonstrates that aPDL1-P-DOX treatment induces a profound reprogramming of the tumor transcriptome. The therapy effectively promotes a potent Th1-type immune response, characterized by the upregulation of chemokines that recruit effector immune cells and the activation of central inflammatory pathways like NF-κB. This shift from an immune-suppressed (“cold”) to an immune-inflamed (“hot”) tumor microenvironment is a key mechanistic insight into the therapeutic efficacy of the aPDL1-P-DOX regimen.

### 2.6 *In vivo* safety evaluation

The biocompatibility of aPDL1-P-DOX was assessed via histological analysis of major organs and blood biochemistry post-treatment. H&E staining revealed no significant pathological abnormalities (Fig. S12). As shown in Fig. S13, liver function markers, including aspartate transaminase (AST), alanine transaminase (ALT), alkaline phosphatase (AKP), blood urea nitrogen (BUN), creatinine (CRE), and urea (UA), were comparable between aPDL1-P-DOX and PBS-treated mice. These findings confirm that aPDL1-P-DOX exhibits favorable biocompatibility while maintaining potent antitumor efficacy.

## 3 Conclusion

The development of aPDL1-P-DOX nanoparticles represents a robust strategy for precise tumor-targeted chemotherapy combined with immunomodulation. Rational design integrated ROS-responsive drug release, PDL1-mediated active targeting, and a self-amplifying positive feedback mechanism. Since IgG-P-DOX possesses identical physicochemical properties (size, zeta potential, ROS-responsiveness) but lacks PDL1-binding capability, the 6.3-fold higher tumor accumulation of aPDL1-P-DOX versus IgG-P-DOX at 24 h (Fig. 2G) represents the specific contribution of PDL1-mediated active targeting beyond passive EPR. In the pre-treatment experiments (Fig. 2H), mice received unlabeled aPDL1-P-DOX (which upregulates PDL1 via DOX release) versus free aPDL1 (which blocks PDL1 without upregulation) or aPDL1+IgG-P-DOX (mixed formulation without covalent conjugation) 24 h prior to Cy5-labeled aPDL1-P-DOX injection. The Pre-aPDL1-P-DOX group showed the strongest fluorescence enrichment, supporting that DOX-mediated PDL1 upregulation (not mere antibody blocking) drives enhanced recruitment. Based on the IgG-P-DOX control data, passive EPR accounts for baseline tumor accumulation, while the additional 5.3-fold increase observed for aPDL1-P-DOX represents PDL1-mediated active targeting. This distinction is further corroborated by the time-dependent amplification of the accumulation ratio (4.2-fold at 3 h increasing to 6.3-fold at 24 h), which is consistent with progressive PDL1 upregulation and feedback-enhanced targeting rather than static EPR-mediated accumulation.

The self-sustaining feedback amplified therapeutic efficacy by upregulating PDL1 on tumor cells, enhancing aPDL1-P-DOX targeting, and increasing intracellular drug uptake. This mechanism synergized with robust ICD induction, evidenced by elevated CRT exposure, HMGB-1/ATP release, and increased pro-inflammatory cytokines (IFN-γ, TNF-α, IL-6, IL-12), effectively remodeling the immunosuppressive tumor microenvironment. Consequently, aPDL1-P-DOX elicited potent antitumor immunity, marked by increased infiltration of CTLs, Ths, and DCs and decreased MDSCs, culminating in a 98.7% tumor inhibition rate and 80% long-term survival in murine models.

Therapeutic superiority of aPDL1-P-DOX was further corroborated by negligible systemic toxicity, as evidenced by histopathological analysis of major organs and biochemical assessments. Collectively, these findings advance the field of nanomedicine by integrating feedback-enhanced tumor targeting with immunogenic chemotherapy, offering a translatable paradigm to overcome biological barriers in cancer therapy. Future investigations will evaluate its combinatorial potential with checkpoint inhibitors or adoptive cell therapies to further potentiate anti-suit immunity. For future clinical translation, several challenges of this nanoparticle system still exist. Its multi-step synthesis impedes GMP-scale production, which can be resolved via optimized batch reactions, continuous flow conjugation and standardized quality control. Murine anti-PDL1 should be replaced by humanized antibodies, and primate immunogenicity tests of poly(L-glutamic acid) are necessary. Tumoral PDL1 heterogeneity demands companion diagnostics for personalized therapy, while co-administration with checkpoint inhibitors, adoptive cell or targeted therapies may further elevate efficacy.

## 4 Experimental section/methods

### 4.1 Materials

DOX·HCl was purchased from Shanghai Macklin Biochemical Technology Co., Ltd (Shanghai, China). DBCO-modified Fc-III-4C peptide (Pep-DBCO) was synthesized by Shanghai GL Biochem Ltd. (Shanghai, China). Anti-mouse PDL1 monoclonal antibody (aPDL1; Clone 10F.2H11, BE0361) was obtained from BioXCell (Lebanon, NH, USA). ELISA kits for mouse IFN-γ, TNF-α, IL-12, and IL-6 came from Thermo Fisher Scientific (Waltham, MA, U.S.A.). Anti-mouse calreticulin (cat # ab196159) was procured from Abcam (Hanam, Republic of Korea). APC anti-PDL1, FITC anti-CD3, PE/Cy7 anti-CD4, APC anti-CD8, PE anti-CD44, APC/Cy7 anti-CD62L, APC/Cy7 anti-CD11b, PE anti-Gr1, APC anti-CD80, and FITC anti-CD11c antibodies were purchased from BioLegend Inc. (San Diego, USA).

### 4.2 Cell lines

Murine colorectal MC38 cells (American Type Culture Collection, ATCC, RRID: CVCL_B288) were cultured in RPMI 1640 medium supplemented with 10% fetal bovine serum (FBS) and 1% penicillin-streptomycin at 37°C with 5.0% CO_2_.

### 4.3 Animals

Female C57BL/6 mice (6-8 weeks old) were purchased from Beijing Vital River Laboratory Animal Technology Co., Ltd. All animal experiments were conducted in accordance with the Guidelines for Care and Use of Laboratory Animals and approved by the Animal Ethics Committee of the Jilin University (approval number: 2025700). All procedures were performed under anesthesia, and all efforts were made to minimize animal suffering.

### 4.4 Preparation and characterization of Peptide-DBCO and aPDL1-DBCO

Peptide-DBCO was synthesized by conjugating Fc-III-4C (2.6 mg, 1.5 μmol) with DBCO-PEG_2k_-NHS (0.2 mg, 1.0 μmol) in anhydrous dimethylformamide (DMF, 1.5 mL) at room temperature for 16 h. The reaction mixture was dialyzed (MWCO = 7000 Da) against ultrapure water for 72 h and subsequently lyophilized to yield Peptide-DBCO as a solid. To prepare aPDL1-DBCO, the synthesized Peptide-DBCO (10.6 mg, 0.1 μmol) was dissolved in Tris-HCl buffer (pH 7.4, 50.0 mM, 1.0 mL) and incubated with aPDL1 to afford the aPDL1-DBCO conjugate. Pep-P-DOX was generated via a copper-free click reaction between Pep-DBCO (10.6 mg, 0.1 mM) and N_3_-P-DOX (1.0 mM) in anhydrous DMF (1.5 mL) at room temperature for 16 h. The reaction mixture was purified by dialysis (MWCO = 7000 Da) against deionized water, followed by lyophilization to yield Pep-P-DOX. Successful conjugation of Pep-DBCO was confirmed by quantifying elemental sulfur using inductively coupled plasma emission spectrometry.

### 4.5 Synthesis and characterization of N_3_-P-DOX

N_3_-PLG-PBA was synthesized following the author's previous protocol, with an average of 160 PLG repeating units. DOX·HCl (100 mg) was dissolved in deionized water (50 mL), and triethylamine was added to generate the DOX free base, which was extracted three times with chloroform. Residual chloroform and trace triethylamine were removed under vacuum to generate purified DOX free base. N_3_-PLG-PBA was dissolved in DMF and mixed with DOX. The reaction was allowed to proceed at 35°C in the dark for 6 h. The mixture was subsequently dialyzed against deionized water (MWCO = 3500 Da) for 72 h to remove DMF and small-molecule impurities. Lyophilization afforded N_3_-P-DOX with a yield of 56.8%. Particle size was characterized using dynamic light scattering.

### 4.6 Preparation and characterization of aPDL1-P-DOX

Pep-P-DOX (10.0 μM) was dissolved in 0.5 mL sterile PBS (pH 7.4), followed by the addition of aPDL1 (10.0 μM, 0.5 mL). The mixture was stirred at 4°C in the dark for 4 h aPDL1-P-DOX nanoparticles were isolated via ultrafiltration, and their hydrodynamic diameter and zeta potential were analyzed using a Zetasizer Nano ZS (Malvern Panalytical). IgG-P-DOX nanoparticles were prepared using an analogous procedure. For stability assessment, aPDL1-P-DOX was incubated with sterile PBS (1.0 mg/mL), and particle size changes were monitored using a Zetasizer Nano ZS instrument.

### 4.7 In vitro drug release of aPDL1-P-DOX

The release profile of DOX from aPDL1-P-DOX was evaluated in PBS containing different concentrations of H_2_O_2_ (0, 20, and 100 μM). aPDL1-P-DOX (equivalent to 30.0 μg/mL DOX, 2.0 mL) was placed in a dialysis bag (MWCO = 3500 Da) and then immersed in 50.0 mL of release medium at 37°C with gentle horizontal shaking (100 rpm). At predetermined time intervals (0, 0.5, 1, 2, 4, 8, and 24 h), 3.0 mL of dialysate was collected and replaced with an equal volume of fresh media. DOX concentration was determined using ultraviolet/visible (UV/vis) spectroscopy (Lambda 365).

### 4.8 ROS-triggered size change

aPDL1-P-DOX (with an equivalent DOX concentration of 30.0 μg/mL, 2.0 mL) was incubated with 100.0 μM H_2_O_2_ at 37°C for 2 h under horizontal shaking (100 rpm). The resulting particle size was measured by Zetasizer Nano ZS.

### 4.9 In vitro cytotoxicity assays

The in vitro cytotoxicity of DOX, IgG-P-DOX, and aPDL1-P-DOX against MC38 cells was determined using the Cell Counting Kit-8 (CCK-8) assay, following a modified manufacturer's protocol. MC38 cells were seeded in 96-well plates at a density of 5 × 10^3^ cells per well and incubated overnight. Various formulations were then added at DOX concentrations ranging from 0.1 to 5.0 μg/mL, and cells were incubated for 48 h. Subsequently, 20.0 μL of CCK-8 reagent was added per well, and cells were incubated for 1 h. Cell viability was then determined using a microplate reader (TECAN, Infinite M200) at 450 nm.

### 4.10 Cellular PDL1 upregulation determination

MC38 cells (5 × 10^4^) were seeded in 12-well plates and allowed to adhere overnight. Cells were treated with PBS, DOX, aPDL1+DOX, IgG-P-DOX, aPDL1+IgG-P-DOX, or aPDL1-P-DOX at a DOX concentration of 0.5 μg/mL for 24 h. For flow cytometry sample preparation, cell debris and non-cellular events were excluded based on FSC/SSC gating. Then cells stained with APC-conjugated anti-PDL1 antibodies for 30 min at room temperature in the dark, washed three times with PBS, and analyzed for PDL1 expression via flow cytometry.

### 4.11 In vitro cellular uptake

MC38 cells were seeded into 12-well plates at a density of 5 × 10^4^ cells per well and incubated overnight. Subsequently, the cells were treated with PBS, DOX, aPDL1+DOX, IgG-P-DOX, aPDL1+IgG-P-DOX, or aPDL1-P-DOX at a final DOX concentration of 0.5 μg/mL for 24 h. Following treatment, the cells were fixed with 4% paraformaldehyde and stained with Hoechst 33342 for 15 min. Fluorescence imaging was performed using confocal laser scanning microscopy (CLSM).

### 4.12 In vitro induction of immunogenic cell death (ICD)

MC38 cells were seeded into 12-well plates at 5 × 10^4^ cells per well and incubated overnight, followed by treatment with PBS, DOX, aPDL1+DOX, IgG-P-DOX, aPDL1+IgG-P-DOX, or aPDL1-P-DOX at a DOX concentration of 0.5 μg/mL for 24 h. For CRT analysis, the cells were fixed with 4% paraformaldehyde for 15 min and then incubated with anti-Alexa Fluor 647-CRT for 2 h at room temperature. The nuclei were stained with Hoechst 33342 for 10 min. After washing with PBS, fluorescence was visualized via CLSM, and surface CRT expression was quantified using a flow cytometer. Extracellular release of high-mobility group box 1 (HMGB1) was measured in the cell culture medium using an ELISA assay kit (Jianglaibio). Extracellular ATP levels were quantified using an ATP detection kit (Beyotime Biotechnology).

### 4.13 Biodistribution

The *in vivo* biodistribution of aPDL1-P-DOX was evaluated in female C57BL/6 mice bearing subcutaneous MC38 tumors (1 × 10^6^ cells injected in the right flank). When tumor volumes reached approximately 200 mm^3^ (n = 3), mice were intravenously administered IgG-P-DOX or aPDL1-P-DOX at a DOX dose of 10 mg/kg. At 3 and 24 h post-injection, major organs and tumors were excised, weighed, and homogenized in a 1:1 (v/v) PBS-methanol mixture via ultrasonication. Homogenates were centrifuged (2500 × g, 10 min). DOX concentrations in the supernatants were quantified by high-performance liquid chromatography (HPLC). For fluorescence-based tumor targeting assessment, mice were intravenously injected with aPDL1, aPDL1+IgG-P-DOX, or aPDL1-P-DOX at a DOX dose of 10 mg/kg (n = 3). After 24 h, Cy5-labled aPDL1-P-DOX was injected. Major organs and tumor tissues were harvested 2 h later and imaged using an IVIS Lumina Series III system.

### 4.14 Pharmacokinetic profiles

Sprague-Dawley rats (average weight 200 g) received tail vein injections of N_3_-P-DOX or IgG-P-DOX at a DOX dose of 5 mg/kg (n = 3). Blood samples were collected from the orbital venous plexus at predetermined time points. Serum was isolated via centrifugation and mixed with an equal volume of methanol overnight to ensure complete DOX dissolution. Samples were centrifuged (2500 × g, 10 min), and DOX concentrations in the supernatants were measured by HPLC using a standard calibration curve. Half-life of the drug (t_1/2_) was calculated using PKSolver.

### 4.15 *In vivo* antitumor efficacy

A subcutaneous MC38 tumor model was established by injecting 1 × 10^6^ MC38 cells in 100 μL PBS into the right flank of anesthetized C57BL/6 mice. When the tumor volume reached about 100 mm^3^, mice were randomly assigned into five groups (n = 5) and intravenously treated with PBS, aPDL1 (5 mg/kg), IgG-P-DOX (containing 5 mg/kg IgG and 2 mg/kg DOX), aPDL1+IgG-P-DOX (containing 5 mg/kg aPDL1, 5 mg/kg IgG, and 2 mg/kg DOX), or aPDL1-P-DOX (containing 5 mg/kg aPDL1 and 2 mg/kg DOX) on days 0, 3, 6, 9, and 12. Tumor volumes and body weights were measured every 2 days. The tumor volume (V) was calculated as V = length (mm) × width^2^ (mm^2^) × 0.5. Survival was monitored, and mice were euthanized when tumors reached 2000 mm^3^ in accordance with ethical guidelines.

### 4.16 *In vivo* antitumor immune response

Tumor tissues were harvested post-therapy and processed into single-cell suspensions. After cell counting, the cells were stained with fluorophore-conjugated antibodies according to the manufacturer's protocols: FITC anti-CD3, PE/Cy7 anti-CD4, APC anti-CD8, FITC anti-CD11c, APC anti-CD80, APC/Cy7 anti-CD11b, and PE anti-Gr1. Flow cytometry gating identified cytotoxic T lymphocytes (CTLs, CD3^+^CD8^+^), helper T cells (Ths, CD3^+^CD4^+^), matured DCs (CD11c^+^CD80^+^), and myeloid-derived suppressor cells (MDSCs, CD11b^+^Gr-1^+^). Splenocytes were also harvested and stained with FITC anti-CD3, PE/Cy7 anti-CD4, APC anti-CD8 PE, anti-CD44, and APC/Cy7 anti-CD62L to quantify CTLs, Ths, central memory T cells (Tcm, CD3^+^CD8^+^CD62L^+^CD44^+^), and effector memory T cells (Tem, CD3^+^CD8^+^CD62L^−^CD44^+^). Tumor tissue cytokine levels—including IFN-γ, TNF-α, IL-6, and IL-12—were quantified using ELISA kits according to the manufacturer's instructions.

### 4.17 Histological analysis

Tumor tissues were collected at the end of the antitumor experiment for histological and immunological evaluation. The analyses included H&E staining, immunofluorescence for TUNEL, CD3, CD4 and CD8, and immunohistochemistry for Ki67 and PDL1.

### 4.18 Bioinformatic analysis

RNA-seq raw reads underwent quality control and adapter trimming using FastQC and Trimmomatic. Clean reads were aligned to the Mus musculus GRCm39 via HISAT2 (v2.2.1), and gene-level counts were obtained with featureCounts (Subread v2.0.3). Differential gene expression analysis was conducted using DESeq2 (v1.38.3), with significantly differentially expressed genes (DEGs) defined as those having an adjusted *p*-value < 0.01​and​|log_2_ fold change| > 1. Downstream analysis and visualization were performed in R (v4.4), utilizing reshape2 (v1.4.4) for data restructuring, ggplot2 (v3.5.2) and ggpubr (v0.6.0) for plotting, and sparklyr (v1.9.0) with sparklyr.nested (v0.0.4) for large-scale matrix operations. Heatmaps, bar plots, and lollipop plots were generated to display gene expression and pathway enrichment outcomes, with publication-grade resolution and standardized styling.

### 4.19 *In vivo* toxicity analysis

At the conclusion of the anti-tumor efficacy experiment, blood samples and major organs were collected for biochemical assessment and histopathological evaluation.

### 4.20 Statistical analysis

All quantitative data are expressed as means ± standard deviation (SD). Comparisons among multiple groups were performed using one-way ANOVA, followed by Tukey's multiple comparison test. A *p*-value < 0.05 was considered statistically significant, with significance levels indicated as follows: *p < 0.05, **p < 0.01, ***p < 0.001, ****p < 0.0001.

## CRediT authorship contribution statement

**Xiaoshuang Wang:** Methodology, Writing – original draft. **Wanze Zhang:** Writing – review & editing. **Chenxi Zhao:** Methodology. **Di Wang:** Resources. **Yingyu Zhang:** Software. **Jianfeng Wang:** Methodology. **Xinyu Li:** Methodology. **Jinbao Wang:** Project administration, Resources. **Xuemei Han:** Funding acquisition. **Fuxin Xue:** Conceptualization, Funding acquisition, Methodology, Writing – review & editing. **Linlin Liu:** Investigation, Methodology, Writing – review & editing. **Zhaohui Tang:** Investigation, Methodology, Visualization.

## Declaration of competing interests

The authors declare no other competing interests.

## Data Availability

Data will be made available on request.
