# Supplementary material for: PDL1-driven self-amplifying drug homing with immune homeostasis restoration
Source: Mater Today Bio. 2026 Aug 10;40:103538. doi: 10.1016/j.mtbio.2026.103538 (PMC13487320; doi:10.1016/j.mtbio.2026.103538)
Supplement: Supplementary file 1 [file mmc1.docx]

Supporting Information

**PDL1-driven self-amplifying drug homing with immune homeostasis restoration**

Xiaoshuang Wang^a#^, Wanze Zhang^b#^, Chenxi Zhao^b^, Di Wang^a^, Yingyu Zhang^a^, Jianfeng Wang^b^, Xinyu Li^d^, Jinbao Wang^b*^, Xuemei Han^a*^, Fuxin Xue^b*^, Linlin Liu^b*^ and Zhaohui Tang^c^

**Figure S1**. ^1^H NMR spectrum of N_3_-PLG-PBA.

**Figure S2**. ^1^H NMR spectrum of N_3_-P-DOX.

**Figure S3**. The size stability of aPDL1-P-DOX in the PBS (pH 7.4) for 6 days.

**Figure S4**. Size changes of aPDL1-P-DOX after incubation with 100 μM H_2_O_2_ for 2 h.

**Figure S5.** Representative gating strategy. Debris was excluded based on forward scatter (FSC) and side scatter (SSC), followed by exclusion of dead cells using a viability dye. Live single cells were subsequently analyzed for PDL1 expression.

**Figure S6.** Assessment of cellular uptake of DOX-based formulations mediated by the positive feedback mechanism of PDL1 blockaed. (scale bar = 50 μm)

**Figure S7.** Relative fluorescence intensity of DOX in cells. Cells were treated with different formulations, and the intracellular DOX fluorescence was captured using a confocal microscope and quantified using ImageJ software on 16-bit TIF images. Data are presented as mean ± SD (n = 3).

**Figure S8**. Pharmacokinetics of the clearance of IgG-PLG-DOX and N_3_-P-DOX (n = 3).

**Figure S9.** Quantitative analysis of (A) HSP70 and (B) HSP90 levels in tumor tissues from mice receiving different treatments. Data are presented as mean ± SD (n = 3).

**Figure S10**. Representative flow cytometry plots indicating the proportions of (A) CTLs (CD3^+^CD8^+^), (B) Ths (CD3^+^CD4^+^), (C) DC cells (CD11c^+^CD80^+^), and (D) NK cells (CD11b^+^Gr-1^+^) in tumors after different treatment for 20 days.

**Figure S11**. Representative flow cytometry plots indicating the proportions of (A) CTLs (CD3^+^CD8^+^), (B) Ths (CD3^+^CD4^+^), (C) Tcm (CD3^+^CD8^+^CD62L^+^CD44^+^), and Tem (CD3^+^CD8^+^CD62L^-^CD44^+^) in spleens after different treatment for 20 days.

**Figure S12**. H&E staining images of the major organs after various treatments; scale bars: 100 μm.

**Figure S13**. Concentrations of (A) AKP, (B) ALT, (C) AST, (D) UA, (E) BUN, and (F) CRE in serum were measured at the end of the antitumor efficacy experiment (n = 4).

**Table S1.** List of abbreviations in manuscript.

| Abbreviations | Full name |
| --- | --- |
| ADC | Antibody-drug conjugates |
| PDL1 | Programmed death-ligand 1 |
| DOX | doxorubicin |
| aPDL1-PLG-DOX | aPDL1-P-DOX nanoparticles |
| ROS | Reactive oxygen species |
| CLSM | Confocal laser scanning microscope |
| CRT | Calreticulin |
| ICD | Immunogenic cell death |
| PBS | Phosphate-buffered saline |
| PDI | Polydispersity index |
| DEGs | Differentially expressed genes |
| KEGG | Kyoto encyclopedia of genes and genomes |
| HMGB1 | High-mobility group box 1 |
| Go | Gene ontology |
| CTLs | Cytotoxic T lymphocytes |
| TEM | Transmission electron microscope |
| H&E | Hematoxylin and eosin |
| TUNEL | TdT-mediated dUTP nick-end labeling |
| AST | Aspartate transaminase |
| ALT | Alanine transaminase |
| AKP | Alkaline phosphatase |
| BUN | Blood urea nitrogen |
| CRE | Creatinine |
| UA | Urea |
| DMF | N,N-Dimethylformamide |

**Table S2.** The antibodies indicating suppliers and titers in manuscript.

| Antibody | Item Number | | Indicating suppliers | Titers |
| --- | --- | --- | --- | --- |
| APC anti-mouse PDL1 antibody | 124312 | BioLegend | | 1:200 |
| FITC anti-mouse CD3ε antibody | 100204 | BioLegend | | 1:200 |
| PE anti-mouse Gr-1 Antibody | 108408 | BioLegend | | 1:200 |
| APC anti-mouse CD80 antibody | 104718 | BioLegend | | 1:200 |
| APC anti-mouse CD8 antibody | 100712 | BioLegend | | 1:200 |
| PE/Cy7 anti-mouse CD4 antibody | 100422 | BioLegend | | 1:200 |
| APC/Cy7 anti-mouse CD62L | 104428 | BioLegend | | 1:200 |
| FITC anti-mouse CD11c antibody | 117306 | BioLegend | | 1:200 |
| InVivoMab anti-mouse PD-L1 | BE0101 | **Bio X Cell** | | 1:5 |
